# Redox metabolic disruptions in the honey bee brain following acute exposure to the pyrethroid deltamethrin

**DOI:** 10.1038/s41598-025-14089-7

**Published:** 2025-08-03

**Authors:** Máté Mackei, Fanni Huber, Barnabás Oláh, Zsuzsanna Neogrády, Gábor Mátis

**Affiliations:** 1 Division of Biochemistry, Department of Physiology and Biochemistry, University of Veterinary Medicine Budapest, Istvan Street 2, Budapest, 1078 Hungary; 2 National Laboratory of Infectious Animal Diseases, Antimicrobial Resistance, Veterinary Public Health and Food Chain Safety, University of Veterinary Medicine Budapest, Istvan Street 2, Budapest, 1078 Hungary

**Keywords:** Molecular apitoxicology, Neurooxidative stress, Pyrethroid, Deltamethrin, Glutathione, Malondialdehyde, Reactive oxygen species, Honey bee, Metabolism, Animal physiology, Entomology

## Abstract

Deltamethrin is a widely used pyrethroid insecticide that has detrimental effects on the redox homeostasis of honey bee (*Apis mellifera*) brains. The decline of pollinating insect populations, including honey bee colonies, is a growing global concern. This problem results in serious ecological and economic concerns as well as veterinary- and animal health-related issues. In addition, exposure to agricultural pesticides is one of the major contributing factors. Adult worker honey bees were exposed to three sublethal concentrations of orally administered deltamethrin (1.975, 3.95, and 7.9 ng/bee/day; corresponding to LD50/40, LD50/20, and LD50/10) for 48 h. In this study, various redox markers, including glutathione concentrations, antioxidant enzyme activities, total antioxidant capacity, hydrogen peroxide concentrations and lipid peroxidation products, were monitored in brain homogenate samples from honey bees. The results revealed significant changes related to the glutathione system, as indicated by decreases in the GSH/GSSG ratio and GSH concentration in all treatment groups. The activities of the monitored enzymes, such as glucose-6-phosphate dehydrogenase (G6PDH), superoxide dismutase (SOD), and xanthine oxidase (XO), were significantly decreased, highlighting the altered function of the enzymatic antioxidant defense system. Moreover, pronounced lipid peroxidation was detected in the highest-dose group, as indicated by increased malondialdehyde (MDA) levels; however, H_2_O_2_ levels were unchanged, suggesting the effective activation of ROS-scavenging adaptation mechanisms. The present study provides valuable insights into the molecular mechanisms of deltamethrin toxicity in honey bees, suggesting that redox metabolism is negatively affected. Understanding the exact mechanism of action may contribute to the identification of new possibilities for effective intervention in cellular metabolic processes in the future through the targeted use of novel protective feed additives or other methods, which are of particular importance for animal health as well as for this field of veterinary medicine.

## Introduction

The decline in pollinating insect species is a growing problem worldwide. One of the most well-known examples of this is colony collapse disorder (CCD), a multifactorial disease affecting honey bees that can be attributed to several causes^1^. In recent years, several studies have demonstrated the importance of infectious diseases and the role of environmental stressors, such as poor foraging conditions and nutritional disorders, an unpredictable climate, and other causes, e.g. exposure to electromagnetic radiation and migratory beekeeping-related stress^2^. However, in addition to these stressors, one of the most significant factors is likely exposure to pesticides widely applied in modern agriculture^3^. Among these pesticides, insecticides such as deltamethrin may be of particular importance. Deltamethrin is an effective agent in current agricultural practices for protecting many crops, such as wheat, maize, oilseed rape, and sunflower, from insect pests^4^. It belongs to the group of pyrethroids, which are synthetically produced compounds with a structure similar to that of pyrethrins found in nature. Their stability and efficacy are superior to those of naturally occurring forms, but this can often lead to increased risks to wildlife^5^. Compared to organophosphate and carbamate pesticides, pyrethroid insecticides are highly effective and have more favorable toxicology profiles, which is why they have been widely used since the 1980s. Currently, pyrethroid pesticides play an important role in crop protection, since they constitute approximately 20% of all pesticide sales globally^6^. Pyrethroid agents can be classified into two different classes based on their chemical structure and toxicological profile. The alpha-cyano moiety is absent from type I pyrethroid pesticides, whereas type II pyrethroid pesticides (including deltamethrin) incorporate an alpha-cyano moiety at the phenyl benzyl alcohol position^7^.

The extensive application of pyrethroids in agricultural practices poses significant risks to nontarget insects, particularly pollinators such as *Apis mellifera*, *Bombus spp*., and various solitary and stingless bee species^8^. Acute toxicity results from direct exposure to high concentrations of pyrethroids, typically through oral or topical contact with treated foliage, flowers, pollen, or nectar^9^. This exposure may lead to neurotoxicity, characterized by prolonged activation of voltage-gated sodium channels, causing continuous neuronal excitation, muscular tremors, or paralysis due to an increase in sodium permeability of the neuronal membrane during the rising phase of the action potential^5,6^. Chronic toxicity may also result from sustained exposure to sublethal doses of pyrethroids, leading to reduced cognitive and motor functions, which hinder the ability to locate and gather floral resources, thereby diminishing colony nutrition^10,11^. Additionally, these bees exhibit navigational disorientation, resulting in increased incidences of forager loss and a decreased colony workforce^9^. Sublethal effects may include compromised immune responses, reduced reproductive success, and altered communication behavior^12^.

On the other hand, it is not yet clear which exact causes may result in the above mentioned consequences. It is strongly suggested that increased production of free radicals and oxidative distress may be important factors in the occurrence of these symptoms; however, this hypothesis has not yet been thoroughly investigated in honey bees^13^. Therefore, in the present study, measurements of various oxidative parameters following deltamethrin exposure were aimed to be carried out, with a special emphasis on the glutathione system, which plays a central role in cellular redox balance and detoxification, total antioxidant capacity (TAC), as a general integrative marker, and enzymes such as superoxide dismutase (SOD) and glucose-6-phosphate dehydrogenase (G6PDH) involved in antioxidant defense mechanisms. Additionally, we monitored molecules produced under intense oxidative stress, including malondialdehyde (MDA), a major marker of lipid peroxidation and hydrogen peroxide (H_2_O_2_), one of the most prominent reactive oxygen species (ROS) in the brains of honey bees. The oxidative stress biomarkers selected in this study serve as important indicators of cellular redox status and oxidative damage. Alterations in these markers can reveal disruptions in antioxidant defense mechanisms and the extent of oxidative injury induced by pesticide exposure, thus providing mechanistic insight into the sublethal effects of deltamethrin on honey bee physiology. Given the implications of these findings for pollinator health, such results further underscore the urgent need for pollinator-friendly measures in agriculture. Implementing integrated pest management and adopting strategies that minimize risks to pollinators are essential to sustain both biodiversity and crop production.

## Materials and methods

### Collection, housing and treatment

Forager worker bees were collected from honey and pollen frames in the morning, as described in previous studies^14,15^. The bees used were sourced from a single colony in Veszprém County, Hungary (47° 18’ 32’’ N, 17° 36’ 15’’ E), to ensure consistent health conditions and genetic backgrounds across all the treatment groups. The colony was not subjected to any treatments for 90 days prior to the study, and regular veterinary inspections were carried out to confirm the disease-free status of the colonies. Four treatment groups were randomly assigned, with 3 replicate cages in each group (altogether 12 replicates). The cages measured 30 cm by 20 cm by 20 cm and contained approximately 200 animals each. The bees were kept in a dark room in a controlled environment at 25 ± 1 °C and 60% relative humidity (RH). Honey bees had *ad libitum* access to drinking water and a 50% w/v sucrose solution throughout the study. Prior to treatment, a 36-hour accommodation period was included, during which nothing was administered except the pure feeding solution and water.

The treatment phase of the study lasted for 48 h, during which the feeding solutions were replaced every 8 h. The sucrose solution of the treatment groups was supplemented with deltamethrin (Cat. No.: 45423; purity ≥ 98.0%) according to the following scheme: the “Delta1”, “Delta2”, and “Delta3” groups received concentrations of 48.125 µg/L, 96.25 µg/L, and 192.5 µg/L, respectively. The feeding solutions were prepared as follows: first, 7.5 mg of deltamethrin was dissolved in 100 µl of DMSO. This stock solution was then diluted 1,000-fold, and the calculated volume was subsequently added to the feeding solutions as described above. The final concentration of DMSO in the feeding solutions did not exceed 0.001%. The same amount of DMSO was also added to the control solutions to ensure consistency across all experimental groups.

Control group continuously received sucrose solution during the study. The daily consumption of the feeding solution was considered to be 40 µL per bee, in accordance with our previous studies and available data^15–18^. Therefore, the applied doses corresponded to *per os* lethal dose 50 (LD50)/40 (“Delta1”: 1.975 ng/bee/day), LD50/20 (“Delta2”: 3.95 ng/bee/day) and LD50/10 (“Delta3”: 7.9 ng/bee/day), respectively. Treatments included acute sublethal doses of deltamethrin^19–21^. Mortality was monitored at 12-hour intervals and did not exceed 2% of the total number of bees.

### Sample preparation

Following the treatment period, the bees were placed on dry ice and euthanized prior to transport to the laboratory, where they were stored at − 80 °C. In total, 40 bees (*n* = 10 per treatment group) were randomly selected and dissected for further measurements on ice under a stereomicroscope. The collected tissue samples included the protocerebrum, deutocerebrum, tritocerebrum and subesophageal ganglions. A Potter-Elvehjem homogenizer containing Tissue Protein Extraction Reagent (T-PER) supplemented with 1% Pierce Protease Inhibitor Cocktail (Thermo Fisher Scientific, Waltham, MA, USA), was used for homogenization. The samples were centrifuged at 5000×*g* for 10 min, and the supernatants were used for subsequent measurements.

### Measurements

Reagents used for the measurements were purchased from Merck KGaA (Darmstadt, Germany), unless otherwise indicated. Colorimetric tests were performed using a Multiskan GO 3.2 reader (Thermo Fisher Scientific, Waltham, MA, USA).

#### Total protein concentration

The total protein concentration of each homogenate sample was determined using a colorimetric Pierce™ Bicinchoninic Acid Protein Assay (Cat. No.: 23225; Thermo Fisher Scientific, Waltham, MA, USA) to ensure the same diluted total protein concentration after sample preparation and homogenization. Absorbance values were recorded at 562 nm after 30 min of incubation at 37 °C.

#### Total glutathione, oxidized (GSSG), and reduced (GSH) glutathione levels

The total glutathione and GSSG concentrations were also determined using a colorimetric assay (Cat. No.: 38185). Standards or samples were mixed with buffer solution and incubated for 1 h at 37 °C. The substrate solution, coenzyme solution, and enzyme mixture were subsequently added to each well. For the GSSG measurement, a masking reagent was also included. The absorbance was measured after a 10-minute incubation at 412 nm. The GSH concentration was determined according to the manufacturer’s instructions based on the total glutathione and GSSG values.

#### Glucose-6-phosphate dehydrogenase (G6PDH) activity

To measure G6PDH activity, standards and samples were pipetted into transparent plates, followed by a master reaction mixture containing G6PDH assay buffer, G6PDH developer solution, and G6PDH substrate solution (Cat. No.: MAK015). The plates were incubated at 37 °C, and the initial absorbance values were recorded after 3 min at 450 nm. Measurements were taken every 5 min until the absorbance of the most active sample exceeded that of the highest standard. Enzyme activity was calculated using the formula provided by the manufacturer.

#### Superoxide dismutase (SOD) activity

To determine SOD activity, samples and blanks were added to 96-well plates, followed by the addition of a working solution and enzyme mixture (Cat. No.: 19160). After incubation for 20 min at 37 °C, absorbance was measured at 450 nm. Enzyme activities were calculated according to the formula provided in the assay’s instructions.

#### Xanthine oxidase (XO) activity

Colorimetric tests were also used to measure XO enzyme activity (Cat. No.: MAK078). The reaction mixture consisted of 44 µL o xanthine oxidase assay buffer, 2 µL peroxidase substrate, 2 µL enzyme solution and 2 µL of substrate mixture, which were added to 50 µL samples or standards on a 96-well microplate, as described by the manufacturer. The absorbance values were detected at 570 nm at 5-minute intervals until the value of the most active sample exceeded that of the highest standard.

#### Malondialdehyde (MDA) concentration

The concentration of MDA, a primary marker of lipid peroxidation, was measured using a thiobarbituric acid reactive substances (TBARS)-based colorimetric assay (Cat. No.: MAK085). The stock solution was mixed with the tissue homogenate supernatants or standard solutions and then incubated for 60 min at 95 °C. The absorbance was measured at 532 nm.

#### H_2_O_2_ (hydrogen peroxide) concentration

The fluorometric-based Amplex Red method was utilized to measure H₂O₂ concentrations. The process involved a 30-minute incubation at room temperature, during which 50 µL of working solution (containing Amplex Red stock solution and horseradish peroxidase [HRP]) was mixed with an equal amount of tissue homogenate (Cat. No.: A22188; Thermo Fisher Scientific, Waltham, MA, USA). Fluorescence detection was carried out using a Victor X2 2030 fluorometer (Perkin Elmer, Waltham, MA, USA).

#### Total antioxidant capacity (TAC)

TAC was measured using a commercial colorimetric assay (Cat. No.: MAK187). The homogenate samples and Trolox standards were mixed with the Cu^2+^-containing working solution. The plates were incubated for 90 min at room temperature, and the absorbance was detected at 570 nm.

### Statistics

Data analysis was conducted using GraphPad Prism 9 software (GraphPad Software Inc., San Diego, CA, USA). The normal distribution of samples and homogeneity of variance were confirmed using the Shapiro‒Wilk test and Levene’s test, respectively. Differences between groups were evaluated by one-way analysis of variance (ANOVA) with Dunnett’s post hoc test for pairwise comparisons. Statistical significance was defined as *P* < 0.05. The heatmap was generated with MetaboAnalyst 6.0 (https://www.metaboanalyst.ca). Statistical groups consisted of *n* = 10 randomly selected animal samples from the 3 replicate cages of each treatment for every tested parameter.

## Results

**Fig. 1:**
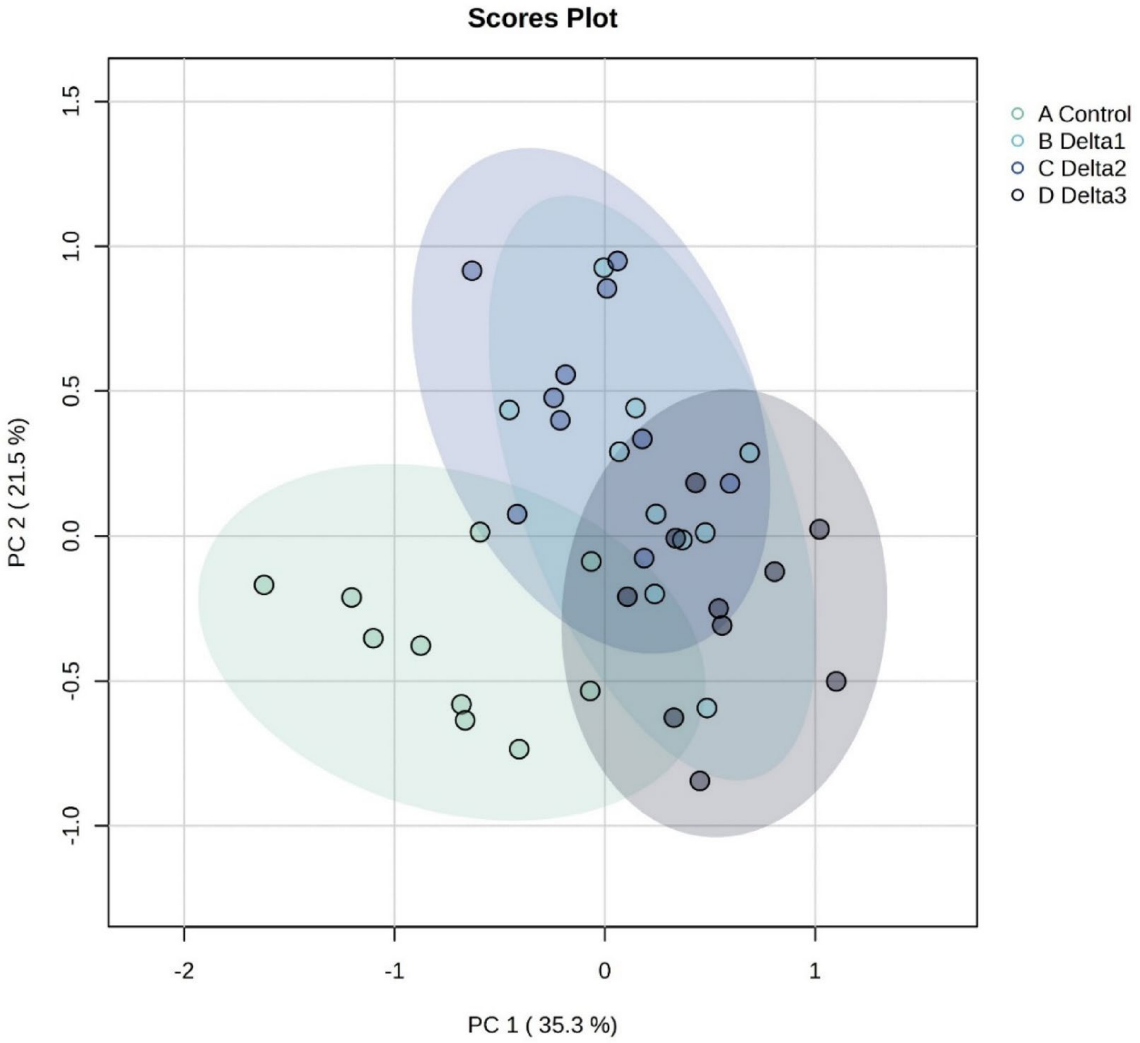
Principal component analysis (PCA) of the measured redox parameters in honey bee brains. In the figure, each dot corresponds to a single sample, with distinct colors used to delineate the respective treatment conditions. “Control” refers to the control group with no treatment; “Delta1”, “Delta2”, and “Delta3” refer to *per os* exposure to 1.975, 3.95 and 7.9 ng/bee/day deltamethrin, respectively.

**Fig. 2:**
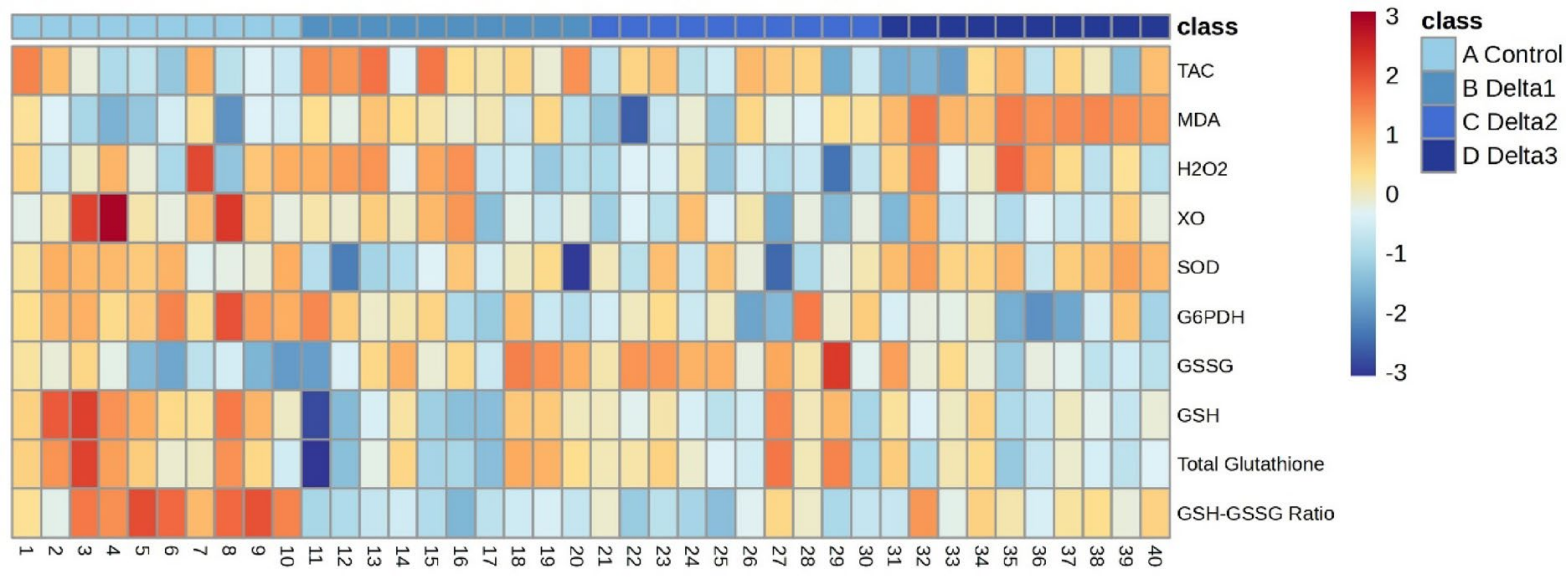
Heatmap representation of redox homeostasis-related parameters across individual samples. The heatmap was constructed using autoscaled data, with color gradients indicating relative concentration levels for each treatment group. Individual samples are displayed along the horizontal axis and are numerically labeled, while treatment groups are distinguished by color-coded annotations. “Control” refers to the control group with no treatment; “Delta1”, “Delta2”, and “Delta3” refer to per os 1.975, 3.95, and 7.9 ng/bee/day deltamethrin, respectively. GSH/GSSG—ratio of reduced to oxidized glutathione; GSH—reduced glutathione; GSSG—oxidized glutathione; G6PDH—glucose‒6‒phosphate dehydrogenase; SOD—superoxide dismutase; XO—xanthine oxidase; H2O2—hydrogen peroxide; MDA—malondialdehyde; TAC—total antioxidant capacity.

**Fig. 3:**
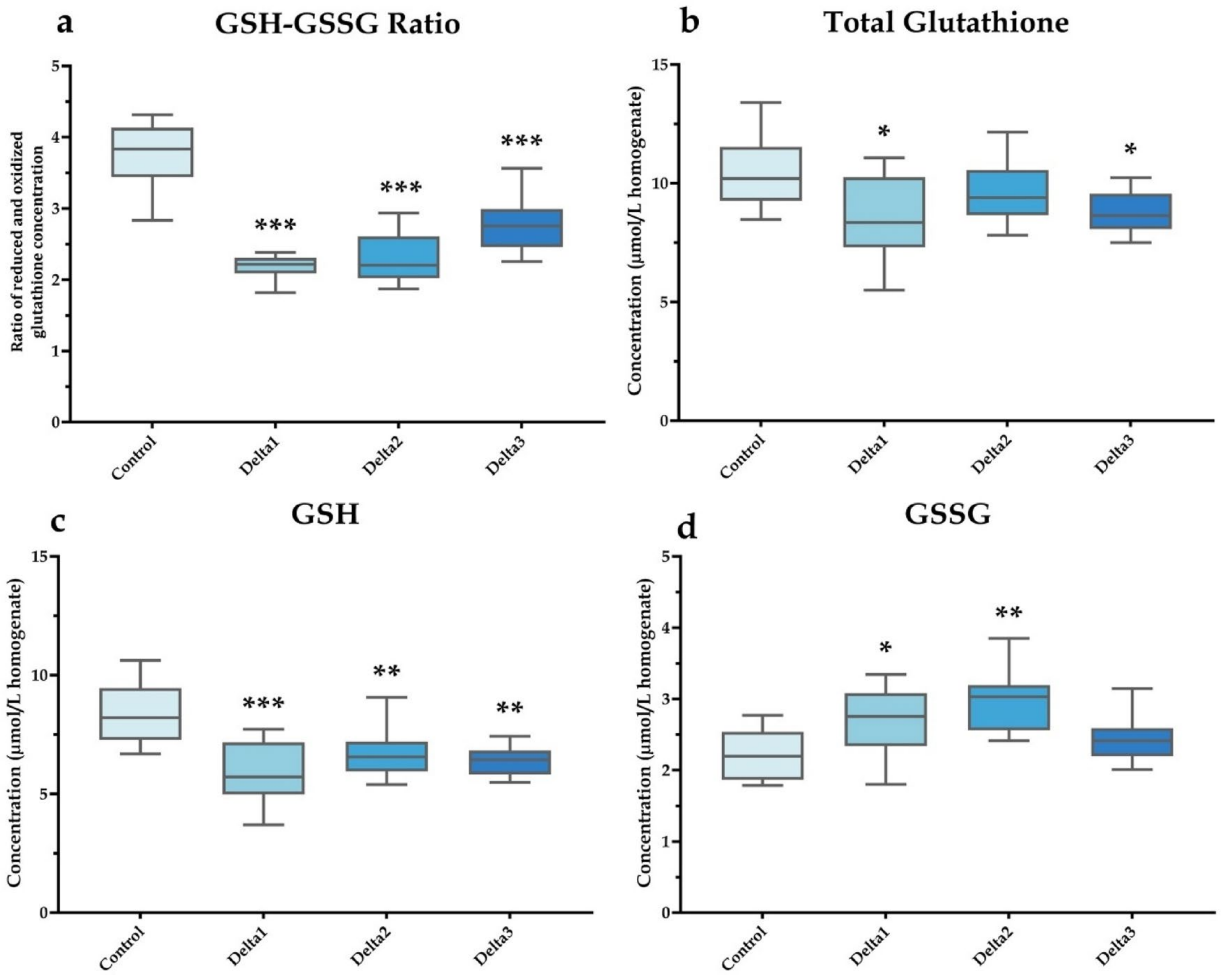
Glutathione levels and ratios in honey bee brain samples. The reduced (GSH) and oxidized (GSSG) glutathione ratios (**a**) and total glutathione (**b**), GSH (**c**) and GSSG (**d**) concentrations were measured. Data are presented as box plots, where whiskers represent minimum and maximum values, boxes indicate upper and lower quartiles, and lines denote medians. “Control” refers to the control group with no treatment; “Delta1”, “Delta2”, and “Delta3” refer to exposure to 1.975, 3.95, and 7.9 ng/animal/day deltamethrin exposure, respectively. Significant differences compared with the control are marked by asterisks. *n* = 10/treatment. *P < 0.05, **P < 0.01, ***P < 0.001.

To provide a comprehensive visualization of the impact of deltamethrin treatment on the redox state of honey bee brains, principal component analysis (PCA) was performed (Fig. 1). PCA of the redox-related variables showed that the first two principal components accounted for approximately 56.8% of the total variance in the dataset (PC1: 35.3%, PC2: 21.5%). These results indicate that deltamethrin exerts a substantial, dose-dependent influence on the redox parameters assessed. Multivariate analysis of variance (MANOVA) using Wilks’ lambda confirmed that deltamethrin exposure significantly affected the overall redox profile of honey bee brains (Λ = 0.0198, F(24, 61.51) = 7.35, *P* < 0.001). This supports the group separation observed in the PCA plot. In order to enhance clarity and provide a comprehensive overview of individual samples, the data are also presented as a heatmap (Figs. 2, 3).

**Fig. 4:**
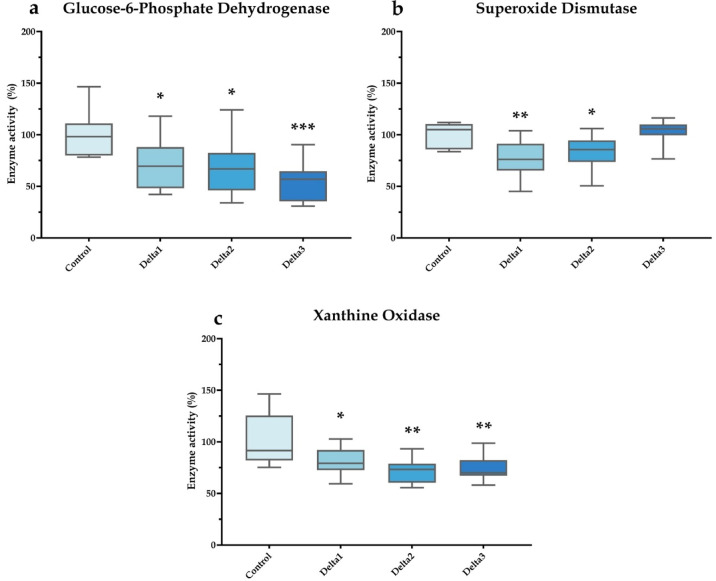
Redox balance-related enzyme activities in honey bee brain samples. Glucose-6-phosphate dehydrogenase (**a**), superoxide dismutase (**b**) and xanthine oxidase (**c**) activities are shown. Data are presented as box plots, where whiskers represent minimum and maximum range values, boxes indivate upper and lower quartiles, and lines denote medians. “Control” refers to the control group with no treatment; “Delta1”, “Delta2”, and “Delta3” refer to 1.975, 3.95, and 7.9 ng/animal/day deltamethrin exposures, respectively. Significant differences compared with the control are marked by asterisks. *n* = 10/treatment. *P < 0.05, **P < 0.01, ***P < 0.001.

 The activity of G6PDH decreased at every applied concentration of deltamethrin applied (*P* = 0.021, *P* = 0.011, and *P* < 0.001, respectively), whereas SOD activities were significantly lower following Delta1 and Delta2 treatments (*P* = 0.003 and *P* = 0.048, respectively). XO activity decreased following Delta1 (*P* = 0.0348), Delta2 (*P* = 0.002), and Delta3 (*P* = 0.004) exposure (Fig. 4).

**Fig. 5:**
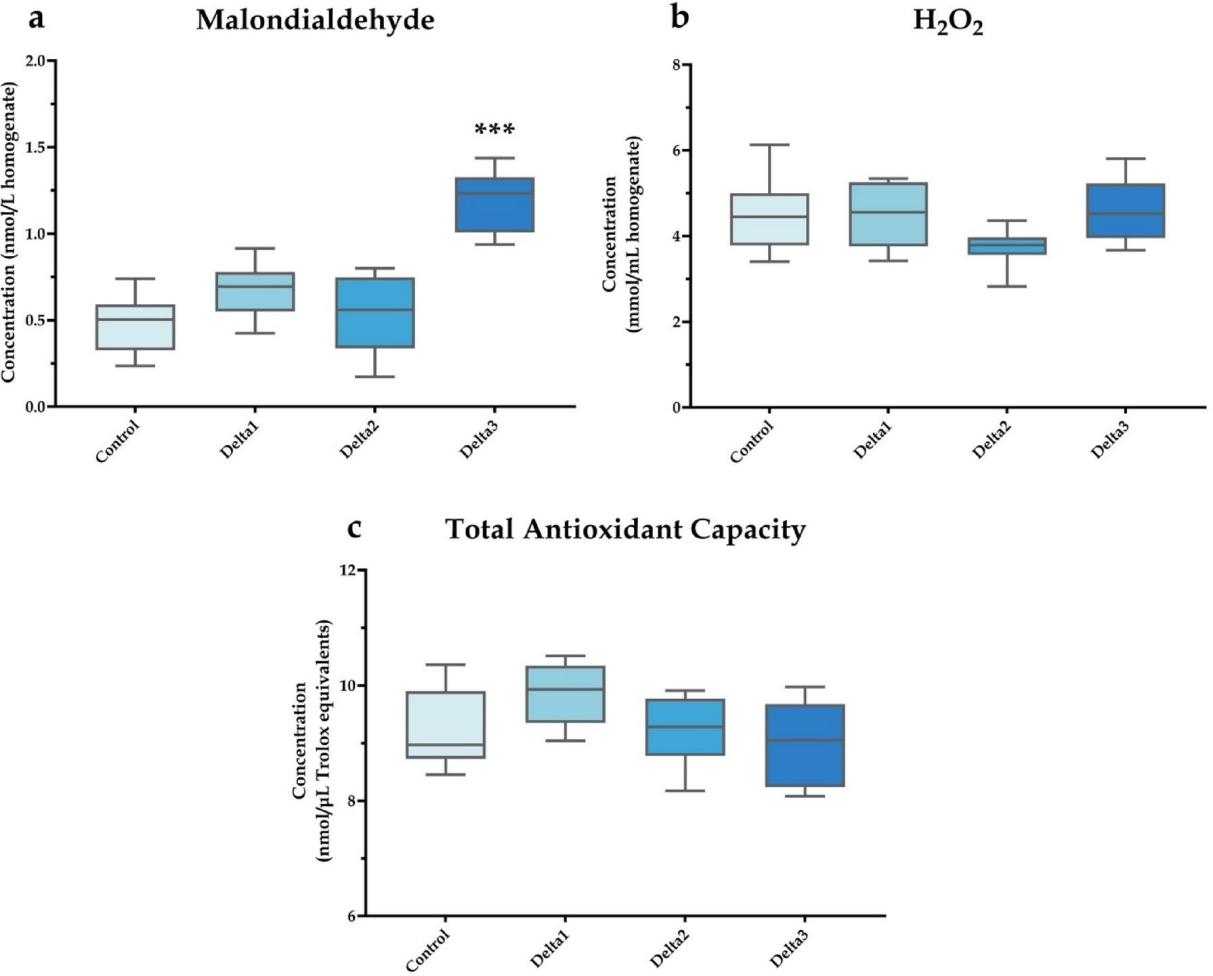
Redox parameters in honey bee brain samples. Malondialdehyde levels (**a**), hydrogen peroxide concentrations (**b**) and total antioxidant capacity values (**c**) are shown. Data are presented as box plots, where whiskers represent minimum and maximum range values, boxes indicate upper and lower quartiles, and lines denote medians. “Control” refers to the control group with no treatment; “Delta1”, “Delta2”, and “Delta3” refer to 1.975, 3.95, and 7.9 ng/animal/day deltamethrin exposures, respectively. Significant differences compared with the control are marked by asterisks. *n* = 10/treatment. ***P < 0.001.

 MDA level in brain samples increased following Delta3 treatment (*P* < 0.001), while no change in H_2_O_2_ concentration or TAC was detected (Fig. 5).

##  Discussion

 Pesticides, including pyrethroid insecticides, can contaminate agricultural and urban areas, exposing honey bees to environmental stress and triggering complex physiological responses. Several studies have reported that deltamethrin decreases survival rates and alters behavior, memory, as well as foraging activity in bees, but the exact mechanisms causing these changes remain unclear^22–24^. In this study, the effects of various deltamethrin concentrations on honey bee brains were examined under laboratory conditions to test the hypothesis that oxidative stress may underline these observations. Our results confirmed that deltamethrin exerts a complex negative effect on redox homeostasis, as reflected by changes in several of the measured parameters. This observation further strengthens the hypothesis that a range of agrichemicals can have negative effects on pollinating insects, including negative changes in redox system functioning, which have already been described for other pesticides^12,25–27^.

 Glutathione is a key component of the antioxidant defense system, and in addition to its role in neutralizing reactive compounds and free radicals, it is also an important contributor to multiple metabolic pathways^28,29^. Therefore, the investigation of this tripeptide was particularly relevant to our research. The treatments significantly decreased the amount of GSH in all deltamethrin-exposed groups and increased the concentration of GSSG in the Delta1 and Delta2 groups compared to control. Moreover, total glutathione levels were reduced in the Delta1 and Delta3 groups.

 Research involving neonicotinoid insecticides has also shown a similar reduction in the GSH/GSSG ratio and total glutathione content, which was associated with heightened oxidative stress^30^. Alterations in the GSH/GSSG ratio reflect a shift towards a more oxidized intracellular environment. Such imbalances reduce cellular ROS detoxification capacity, resulting in increased oxidative damage. Disturbances in glutathione homeostasis have been linked to impaired neural function, decreased learning and memory performance, heightened vulnerability to pathogens, and reduced ability to withstand environmental stress^28,29,31^. These observations underscore the central importance of maintaining glutathione balance for overall bee health and resilience, particularly under conditions of agrochemical exposure.

 Deltamethrin has been shown to decrease GSH levels in the cerebrum and whole brain of quails^32,33^, as well as in the striatum and hippocampus of rats^34^ and mice^35^. However, no additional data are available for these parameters in insect species, particularly in honey bees.

 With respect to the GSH/GSSG ratio, a decrease has been reported in various organs of honey bees exposed to different stressors. On the other hand, to date, no information is available concerning the effects of deltamethrin on this parameter in honey bees^30,31,36^.

 The above observations are supported by the fact that the stimulatory effect of deltamethrin on glutathione peroxidase (GPx) enzyme activity has been observed in several studies, resulting in the conversion of GSH to GSSG and therefore shifting their ratio towards GSSG^37–39^. Similar alterations related to the glutathione system were also observed with other pyrethroids, such as dermal cypermethrin exposure or intraperitoneally applied lambda-cyhalothrin, in Wistar rats^13,40^. The importance of investigating the function of the glutathione system is further supported by the role of glutathione S-transferase (GST) as a key component of pyrethroid resistance in several insect species^41^. GST enzymes contribute to pyrethroid tolerance by catalyzing the conjugation of glutathione to pyrethroids, thereby facilitating their degradation and excretion. This phenomenon has been confirmed in various insect species including *Meteorus pulchricornis*^42^, *Nilaparvata lugens*^43^, *Anopheles sinensis*^44^, and honey bees^45–47^. Further studies also revealed a nonmonotonic dose‒response following pyrethroid flumethrin exposure in honey bee larvae, including changes in the enzyme activities of the antioxidant defense system^48^.

 The pentose phosphate pathway is also important in relation to the glutathione system as the produced NADPH + H^+^ is necessary for the reduction of GSSG, by the enzyme glutathione reductase (GR), to GSH. To study this process, we investigated changes in the activity of the key regulatory enzyme G6PDH. According to our findings, deltamethrin significantly decreased G6PDH enzyme activity at every tested concentration, contributing to the impaired GSH/GSSG ratio via the diminished NADPH + H^+^ level. Importantly, according to our recent knowledge, no studies concerning honey bees or other pollinator insects are available in relation to this parameter. Furthermore, since the cytochrome P450 (CYP) enzymes that are directly involved in detoxification reactions also require significant amounts of NADPH + H^+^, a correlation between the two processes has been observed in several studies, demonstrating that the more active G6PDH is, the faster the detoxification processes proceed, which may also be reciprocal^49–51^. Accordingly, the observed decrease in the pentose phosphate pathway may also indirectly slow the detoxification process, thus increasing the detrimental effects of deltamethrin.

 Since SOD is directly connected to the antioxidant defense system due to its important role in the conversion of superoxide anions into molecules of lesser toxicity, its decreased activity in the Delta1 and Delta2 treatment groups is also noteworthy^52^. In honey bees, decreased larval but increased adult SOD activities were observed following pyrethroid lambda-cyhalothrin treatment^47^. Conversely, another study reported increased head and decreased midgut SOD expression but did not analyze activity following flumethrin exposure^27^. However, no studies are available regarding the effects of deltamethrin in honey bees. These findings also highlight that although some compounds may belong to the same chemical group, their effects may vary considerably depending on the given molecule and species-specific variability; moreover, even opposite effects may occur depending on the developmental stage of the animal^52–54^. In addition, studies often show nonmonotonic dose‒response relationship, meaning that the dose dependence cannot be described simply in terms of an increase in concentration leading to increased impairment of the measured parameters^55–57^. This observation is also supported by our results for several parameters, such as the glutathione system and SOD, in which lower concentrations resulted in more significant differences.

 In addition to G6PDH and SOD, deltamethrin also decreased XO activity in our study. The available literature on the effects of pyrethroids on nitrogen metabolism, including XO is still very limited^58–60^. Interestingly, a similar reduction was reported by our research group in a previous experiment in which the effects of the neonicotinoid acetamiprid in honey bees were investigated^15^. While inhibition of XO may decrease H_2_O_2_ levels, it is also important to note that the uric acid produced may also play an important role in the energy supply of insects, particularly in urocytes^61^. On the other hand, as an important buffer and antioxidant molecule, decreased uric acid concentrations resulting from XO inhibition may also negatively affect redox balance^62^.

 Data from the literature suggest that pyrethroid agents increase the concentration of ROS such as H_2_O_2_ which is associated with increased oxidative stress^63,64^. Considering other agents of the same group, it has been described that pyrethroid permethrin increased H_2_O_2_ concentrations in the freshwater amphipod *Echinogammarus tacapensis* after contact exposure^65^ and in *Drosophila melanogaster* following oral treatment^66^. On the contrary, our study revealed no significant increase in H_2_O_2_ levels in honey bee brains, although other redox metabolism-related parameters were affected, suggesting the activation of effective cellular adaptation mechanisms that normalize ROS concentrations.

 Consistent with these observations, deltamethrin increased MDA concentration. This finding suggests that depletion of the antioxidant defense system can ultimately initiate lipid peroxidation, as MDA is a specific marker of the terminal phase of this process^67^. The oxidative breakdown of lipids, especially polyunsaturated fatty acids (PUFAs), constitutes the first step in the chain reaction mechanism of lipid peroxidation. This process damages cells and results in the production of reactive aldehydes, including MDA^68^.

 Consequently, treatment at the highest concentration of deltamethrin resulted in a significant increase in MDA content in our study, indicating intense lipid peroxidation. Although deltamethrin is considered to be one of the least toxic pyrethroids in mammalian species; numerous studies have documented its detrimental effects on these organisms^52^. The effects of environmental deltamethrin exposure on lipid peroxidation have also been reported in earthworms (*Eisenia fetida*)^69^ and in arthropods, such as the Chinese mitten crab (*Eriocheir sinensis*)^70,71^ and the oriental river prawn (*Macrobrachium nipponense*)^72^; however, no change was observed in the black tiger shrimp (*Penaeus monodon*)^73^. It is important to note, that no data are available focusing on the effects of deltamethrin on honey bees. With respect to the broader class of pyrethroids, only limited data exist; for example, flumethrin has been reported to increase MDA concentrations in the heads of adult bees and in larvae^26,27^. In the brains of honey bees, MDA levels may remain elevated as a stable marker of oxidative stress, whereas H_2_O_2_ levels may normalize more rapidly due to the efficient activation of compensatory mechanisms that degrade ROS molecules^28^. However, MDA, as a stable end product of lipid peroxidation, may persist and accumulate for longer periods^67,68^. This discrepancy suggests that while immediate oxidative stressors such as H_2_O_2_ may be rapidly addressed by the antioxidant defense system of honey bees, the MDA-related effects of lipid peroxidation may be more prolonged. Importantly, a key limitation of our experiment is that measurements were made at a single time point, making it difficult to accurately extrapolate the time course of these processes. Additionally, all experimental bees were sourced from a single colony. While this approach allowed for control over genetic and environmental variability, it may limit the generalizability of our findings, as colony-level variation is a recognized factor in honey bee research. Future studies should aim to include multiple colonies to enhance the robustness and applicability of the results.

 In line with the above, no change was observed in TAC, implying that signs of oxidative stress and exhaustion of the glutathione system were present. This observation is further supported by the finding that the total antioxidant capacity of cells is tightly regulated and not easily modulated^74^. Similar results have been reported for pyrethroids in honey bee larvae, where, although flumethrin greatly reduced survival and had a pronounced detrimental effect on several antioxidant parameters, TAC levels were not altered compared to the control group^48^.

##  Conclusion

 Our results demonstrate that deltamethrin exerts broad and significant effects on multiple parameters related to the antioxidant system and oxidative stress. Specifically, activation of the glutathione system, increased lipid peroxidation, and altered activities of several enzymes involved in maintaining the antioxidant balance were observed. However, some stabilizing protective mechanisms may have been initiated in the bee brain, as indicated by unchanged H_2_O_2_ levels, which may reflect compensatory responses, such as the oxidation of GSH to GSSG and consequent depletion of the glutathione system. In summary, these adverse effects underscore the need for careful regulation and the development of pollinator-friendly pest management strategies to safeguard these essential contributors to biodiversity and agricultural productivity. Given the damaging effects of pyrethroids described above, our findings may also support the use of targeted bioactive substances, such as potential feed additives, as preventive and mitigative measures, thus opening novel possibilities in the field of apitoxicology.

## Data Availability

All raw datasets used in the study are available from the corresponding author upon request.

## Abbreviations

GSH/GSSG: Ratio of reduced to oxidized glutathione
GSH: Reduced glutathione
GSSG: Oxidized glutathione
G6PDH: Glucose‒6‒phosphate dehydrogenase
SOD: Superoxide dismutase
XO: Xanthine oxidase
H_2_O_2_: Hydrogen peroxide
MDA: Malondialdehyde
TAC: Total antioxidant capacity
